# GIMAP7 induces oxidative stress and apoptosis of ovarian granulosa cells in polycystic ovary syndrome by inhibiting sonic hedgehog signalling pathway

**DOI:** 10.1186/s13048-022-01092-z

**Published:** 2022-12-30

**Authors:** Anran Xu, Yuanyuan Fan, Song Liu, Lianbing Sheng, Yanyan Sun, Huijun Yang

**Affiliations:** Center of Reproductive Medicine, Maternity and Child Health Care Hospital of Shandong Province/ Key Laboratory of Birth Regulation and Control Technology of the Health Commission of China, 238 Jiangshuiquan Road, Jinan, 250014 Shandong People’s Republic of China

**Keywords:** Polycystic ovary syndrome, GIMAP7, Oxidative stress, Sonic hedgehog signalling pathway

## Abstract

Polycystic ovary syndrome (PCOS) is a gynaecological endocrine disease. The objective of the present study was to investigate the role of GTPase immunity-associated protein (GIMAP) 7 in PCOS. A PCOS rat model was established using dehydroepiandrosterone injection. The data showed that GIMAP7 was mainly located in granulosa cells and was abundantly expressed in the ovarian granulosa cells of PCOS rats. GIMAP7 silencing decreased blood glucose levels, HOMA-IR scores, and number of cystic follicles. In addition, GIMAP7 silencing corrected erratic oestrous cycles, inhibited apoptosis and reduced c-caspase-3 protein expression in the ovarian tissues of PCOS rats. GIMAP7 silencing reduced malondialdehyde (MDA) but increased glutathione (GSH) and superoxide dismutase (SOD) levels in the serum and ovarian tissues of PCOS rats. The effects of GIMAP7 were further investigated in human ovarian granulosa KGN cells. GIMAP7 silencing increased the viability, promoted proliferation, and increased the percentage of S-phase KGN cells. The apoptosis rate was significantly decreased by GIMAP7 silencing. GIMAP7 also inhibited oxidative stress in KGN cells, resulting in decreased levels of reactive oxygen species (ROS) and MDA and increased levels of GSH and SOD. Notably, GIMAP7 inhibited the sonic hedgehog (SHH) signalling pathway, and GIMAP7 silencing increased the expression of the SHH signalling pathway downstream genes *SHH*, *SMO*, and *Gli1.* Inhibition of the SHH signalling pathway using cyclopamine reduced the effect of GIMAP7 silencing on KGN cells. This study proved that GIMAP7 promotes oxidative stress and apoptosis in ovarian granulosa cells in PCOS by inhibiting the SHH signalling pathway.

**Supplementary Information:**

The online version contains supplementary material available at 10.1186/s13048-022-01092-z.

## Highlights

1. GIMAP7 is upregulated in ovary granulosa cells of PCOS rat;

2. GIMAP7 shRNA relieves the symptoms of PCOS rats;

3. GIMAP7 shRNA inhibits the apoptosis and oxidative stress of KGN cells;

4. GIMAP7 inhibits sonic hedgehog signalling pathway in KGN cells.

**Supplementary Information:**

The online version contains supplementary material available at 10.1186/s13048-022-01092-z.

## Introduction

Polycystic ovary syndrome (PCOS), one of the most common reproductive endocrine diseases in women of reproductive age, was first reported in 1935 by Stein and Leventhal [1–3]. PCOS is characterised by polycystic ovaries, ovulatory dysfunction, and hyperandrogenism [4, 5]. PCOS leads to psychological and metabolic disorders, including insulin resistance and type 2 diabetes, and increases the risk of cardiovascular disease and obesity [6–8]. Currently, the clinical drugs used for PCOS treatment include oral insulin sensitisers, antiandrogens, and contraceptives [4, 9, 10]. The potential mechanisms of PCOS have not been fully elucidated, and further exploration of these mechanisms may be conducive to the development of new targets for the treatment of PCOS.

The GTPase immunity-associated protein (GIMAP) gene family consists of eight genes, and seven functional genes: GIMAP1, GIMAP4, GIMAP5, GIMAP6, GIMAP7, GIMAP8, and GIMAP9 are conserved in humans and rats [11–13]. GIMAP7 is a transmembrane protein located in the endoplasmic reticulum and Golgi apparatus [11]. Analysis of GIMAP7 in pan-cancers has shown that GIMAP7 is significantly downregulated in most cancers [14] and its expression is associated with the development and progression of many cancers [15]. For example, GIMAP7 is downregulated in patients with oral cancer [16]. In endometrial cancer, high levels of GIMAP7 expression are conducive to disease-free and overall survival [17]. GIMAP7 expression was lower in head and neck squamous cell carcinoma tumour tissues than in normal tissues [18]. Additionally, GIMAP7 regulates immune infiltration [19, 20]. However, whether GIMAP7 regulates PCOS development remains unclear.

By searching the Gene Expression Omnibus (GEO) database, GIMAP7 was found to be highly expressed in patients with PCOS in the GSE80432 dataset. Therefore, this study aimed to explore the role of GIMAP7 in PCOS. GIMAP7 short hairpin RNA (shRNA) was injected into the ovaries of dehydroepiandrosterone (DHEA)-induced PCOS rats to evaluate the effects of GIMAP7 on PCOS. Additionally, in vitro experiments were performed in human ovarian granulosa cells to confirm the regulatory effect of GIMAP7 on PCOS.

## Material and methods

### Bioinformatic analysis

GIMAP7 expression in patients with and without PCOS was analysed using the GEO dataset GSE80432. The downstream signalling pathways regulated by GIMAP7 were analysed using the Kyoto Encyclopedia of Genes and Genomes (KEGG).

### PCOS rat model

After 1 week of adaptation, twenty-four female Sprague–Dawley rats (3 weeks old, Pengyue Laboratory Animal Breeding Co., Ltd., Jinan, China) were randomly divided into four groups (*n* = 6 per group): Blank, DHEA, DHEA+shNC, and DHEA+shGIMAP7. For PCOS modelling, rats were injected with 60 mg/kg DHEA (Shanghaiyuanye Bio-Technology Co., Ltd., Shanghai, China; dissolved in 0.2 mL of sesame oil) daily for 20 days. Rats in the blank group were injected with the same volume of sesame oil. The experimental protocol of our study was performed in accordance with the Guide for the Care and Use of Laboratory Animals and approved by the Ethics Committee of Maternity and Child Health Care Hospital of Shandong Province (Ethics No. 2021–017).

### Injection of shRNA lentivirus

GIMAP7 shRNA (shGIMAP7, 5′-ACCTCGCTGAACTGGATGATGACTCTTCAAGAGAG AGTCATCATCCAGTTCAGCTT-3′) and shRNA negative control (shNC, 5′-ACCTCGTTCAGGACAGTCGGTAATCTTCAAGAGAGATTACCGACTGTCCTGAACTT-3′) were purchased from Shanghai Genechem Co., Ltd. (Shanghai, China). Twelve PCOS rats aged 5 weeks were divided into two groups (*n* = 6 per group): DHEA+shNC and DHEA+shGIMAP7. Briefly, a small cut was made on PCOS rats to access the ovaries. Lentiviruses carrying shGIMAP7 or shNC (10 μL) were injected into one ovary from a small cut. The microsyringe was held in situ for 5–7 min to prevent lentiviral backflow. Three weeks after the lentivirus injection, experiments were carried out.

### Oestrous cycles

The oestrous cycle comprises four stages: proestrus (P), oestrus (E), metestrus (M), and dioestrus (D). The oestrous cycle was assessed based on the proportion of exfoliated vaginal cells (nucleated epithelial cells, squamous epithelial cells, and leukocytes). Three weeks after lentivirus injection, vaginal smears were obtained for 8 days. Exfoliated vaginal cells were collected using a pipette with PBS and dripped onto glass slides. The proportions of the three cell types were observed under a light microscope (Zeiss, Germany). The oestrous cycle was evaluated as previously described [21] from six rats of each group.

### Glucose tolerance test (GTT)

Twenty days after DHEA injection, the GTT was performed after 16 h of fasting. After fasting, six rats of each group received an intraperitoneal injection of glucose (2 g/kg). Blood glucose levels were measured before glucose injection and at 30, 60, 90, and 120 min after glucose injection using a Glucose Assay Kit (Beyotime Biotechnology, Shanghai, China). Homeostasis model assessment of IR (HOMA-IR) was calculated as follows: $\textrm{HOMA}-\textrm{IR}=\frac{\textrm{fasting}\ \textrm{glucose}\ \left(\textrm{mmol}/\textrm{L}\right)\times \textrm{fasting}\ \textrm{insulin}\ \left(\textrm{mIU}/\textrm{L}\right)}{22.5}$.

### Serum levels of hormones

Blood samples were collected from the abdominal aorta of six rats of each group and were centrifugated at 14000×*g* for 10 min to obtain serum. The serum levels of follicle-stimulating hormone (FSH), luteinizing hormone (LH), insulin, testosterone, and estradiol were measured using a rat FSH ELISA kit (Sango Biotech, Shanghai, China), rat LH ELISA kit (Sango Biotech), rat insulin ELISA kit (Elabscience, Hubei, China), rat testosterone ELISA kit (Sango Biotech), and rat estradiol ELISA kit (Sango Biotech).

### H&E and immunohistochemical (IHC) study

The ovaries were collected from six rats of each group and were fixed with 4% paraformaldehyde, embedded in paraffin, and cut into 5 μm thick slices. After deparaffinization with dimethylbenzene and rehydration with grades of alcohol, the slices were stained with haematoxylin for 10 min and eosin (Beyotime Biotechnology) for 5 min. For IHC, the slices were treated with citrate buffer to retrieve antigens. After incubation with GIMAP7 primary antibody (dilution 1:200, Cat No: 17293–1-AP, Proteintech, Wuhan, China) at 4 °C overnight, the slices were incubated with goat anti-rabbit secondary antibody (dilution 1:500, ab150077, Abcam, Shanghai, China) for 1 h. The results were visualised using a diamino-benzidine kit (Beyotime Biotechnology) and GIMAP7-positive cells were analyzed using ImageJ (National Institutes of Health, Bethesda, MD, USA). IHC score was assigned a 4 tire scoring system, i.e. high positive (3+), positive (2+), low positive (1+) and negative (0).

### TUNEL staining

The apoptosis of ovarian tissues from six rats of each group was assessed by the TUNEL kit (Beyotime Biotechnology). Ovarian slices were de-paraffinised, rehydrated, incubated with proteinase K for 20 min, and washed three times with PBS. The slices were then incubated with 3% H_2_O_2_ solution to inactivate endogenous peroxidase. After washing with PBS, the slices were incubated with TUNEL solution for 1 h and then visualized using with a DAB kit (Beyotime Biotechnology).

### qRT-PCR

Total RNA from ovarian tissues and KGN cells was extracted using the TRIzol reagent (Beyotime Biotechnology). The PrimeScript RT reagent Kit (TaKaRa, Dalian, China) was used to reverse-transcribe RNA to cDNA, and the SYBR qPCR Mix kit (Beyotime Biotechnology) was used to perform qPCR. The mRNA expression was analysed using the 2^–ΔΔCT^ method. GAPDH was used as the internal control. The primer sequences are listed as follows. Rat GIMAP7: 5′-CTGTCCTGCAGAAGTCAAGGC-3′ (forward), 5′-AAGGACACACCGGCTGATTT-3′ (reverse); rat GAPDH, 5′-TGATTCTACCCACGGCAAGTT-3′ (forward), 5′-TGATGGGTTTCCCATTGATGA-3′ (reverse); human GIMAP7: 5′-GCAACAGCTCAAGCAGCCTC-3′ (forward), 5′-AGGCACGTACAAGACCTTCTCT-3′ (reverse); human SHH, 5′-AGCTCTCCAGGCTTGCTACC-3′ (forward), 5′-CGCCACCGAGTTCTCTGCTT-3′ (reverse); human SMO, 5′-GTCGGGCCTCCGGAATG-3′ (forward), 5′-GTCTCATTGGAGGTGGGCTC-3′ (reverse); human Gli1, 5′-GGTCCTGGGGGTGCAATAA-3′ (forward), 5′-AAGAAAAGAGTGGGCCTCTGTC-3′ (reverse); human GAPDH, 5′-GAATGGGCAGCCGTTAGGAA-3′ (forward), 5′-GAGGGATCTCGCTCCTGGAA-3′ (reverse). The experiments were repeated three times in triplicate.

### Western blot

Total protein content in ovarian tissues and KGN cells were extracted using lysis buffer (Beyotime Biotechnology). The protein concentration was assessed using a BCA kit (Beyotime Biotechnology). Then, the protein samples were separated by SDS-PAGE and transferred onto PVDF membranes, followed by incubation with primary antibodies against GIMAP7 (dilution 1:2000, Cat No: 17293–1-AP, Proteintech), c-caspase-3 (dilution 1:1000, ab32042, Abcam), SHH (dilution 1:500, ab135240, Abcam), SMO (dilution 1:1000, ab235183, Abcam), and Gli1 (dilution 1:500, ab134906, Abcam) overnight at 4 °C. After incubation with goat anti-rabbit (dilution 1:1000, ab7090, Abcam) and goat anti-mouse (dilution 1:2000, ab96879, Abcam) secondary antibodies for 2 h, the bands were visualised using a chemiluminescence ECL kit (Beyotime Biotechnology).

### Cell culture

The human ovarian granulosa cell line, KGN (Procell, Wuhan, China), was cultured in Dulbecco’s Modified Eagle Medium (DMEM; Invitrogen, Carlsbad, CA, USA) containing 10% fetal bovine serum (FBS; Hyclone, Logan, UT, USA) and 1% penicillin-streptomycin solution (Invitrogen) at 37 °C. To inhibit the sonic hedgehog (SHH) signalling pathway, 5 μM cyclopamine (MedChemExpress, Monmouth Junction, NJ, USA), an inhibitor of the SHH signalling pathway, was used to treat KGN cells for 24 h [22].

### Cell transfection

GIMAP7 siRNAs (siRNA-1: 5′-ACCTCGAATTGCTGCCCAAGCTGTTATCAAGAGTA ACAGCTTGGGCAGCAATTCTT-3′; siRNA-2: 5′-CAAAAAGAATTGCTGCCCAAGCTGTT ACTCTTGATAACAGCTTGGGCAGCAATTCG-3′) and siRNA negative control (siNC, 5′-ACCTCGCGGCTCTTACACGATATTGATCAAGAGTCAATATCGTGTAAGAGCCGCTT-3′) were synthesised by GeneCopoeia. GIMAP7 siRNAs (20 μM) or siNC were transfected into KGN cells using Lipofectamine 2000 (Invitrogen). After 48 h of transfection, subsequent experiments were performed.

### CCK-8 assay

Cell viability was determined using a CCK-8 kit (Beyotime Biotechnology). KGN cells (4 × 10^3^ cells/well) were seeded in 96-well plates and incubated for 24 h. After transfection with GIMAP7 siRNAs or siRNA negative control for 48 h, KGN cells were incubated with CCK-8 solution for 2 h at 37 °C. Absorbance (450 nm) was measured using a microplate reader (Thermo Fisher Scientific, Waltham, MA, USA). The experiments were repeated three times in triplicate.

### EdU staining

The proliferation of KGN cells was measured using an EdU kit (Beyotime Biotechnology). KGN cells (5 × 10^4^ cells/well) were seeded in 24-well plates and incubated for 24 h. After transfection with GIMAP7 siRNAs or siNC for 48 h, KGN cells were incubated with EdU solution for 4 h at 25 °C and fixed for 15 min. KGN cells were then stained with the Apollo dye reagent for 30 min and DAPI for 5 min. The results were observed under a fluorescence microscope. The experiments were repeated three times in triplicate.

### Flow cytometry

After GIMAP7 siRNA transfection for 48 h, KGN cells were collected, washed twice with cold PBS, and then stained with FITC and PI (Beyotime Biotechnology) for 20 min in the dark. Apoptotic cells were analysed using flow cytometry and FlowJo software (Tree Star Corp). For cell cycle analysis, KGN cells were fixed in 70% ethanol overnight at 4 °C, followed by incubation with PI for 20 min. The cell cycle was analysed using Modfit LT software and flow cytometry. The experiments were repeated three times in triplicate.

### Reactive oxygen species (ROS) generation

ROS generation in KGN cells was analysed using a ROS assay kit (Beyotime Biotechnology). After GIMAP7 siRNAs transfection for 48 h, the KGN cells were stained with DCFDA for 20 min. After washing with PBS, ROS generation was observed under a fluorescence microscope. The mean fluorescence intensity of DHE was quantified using ImageJ (National Institutes of Health). The experiments were repeated three times in triplicate.

### Oxidative stress detection

Ovarian tissues and KGN cells were homogenised (for ovarian tissues) or lysed (for KGN cells). Ovarian tissues and KGN cells were centrifuged for 10 min at 10,000×*g* to collect supernatants. Malondialdehyde (MDA), superoxide dismutase (SOD), and glutathione (GSH) levels were measured using the Total Superoxide Dismutase Assay Kit with NBT, Lipid Peroxidation MDA Assay Kit, and Glutathione Assay Kit (Solarbio, Beijing, China), respectively. The experiments were repeated three times in triplicate.

### Statistical analysis

The results were analysed using GraphPad Prism 7.0 (San Diego, CA, USA), and are shown as means ± SD. Differences between two groups were analysed using Student’s t-test. Differences among multiple groups were analysed using a one-way or two-way analysis of variance. *P* < 0.05 was considered statistically significant.

## Results

### GIMAP7 expression is upregulated in PCOS rat

**Fig. 1:**
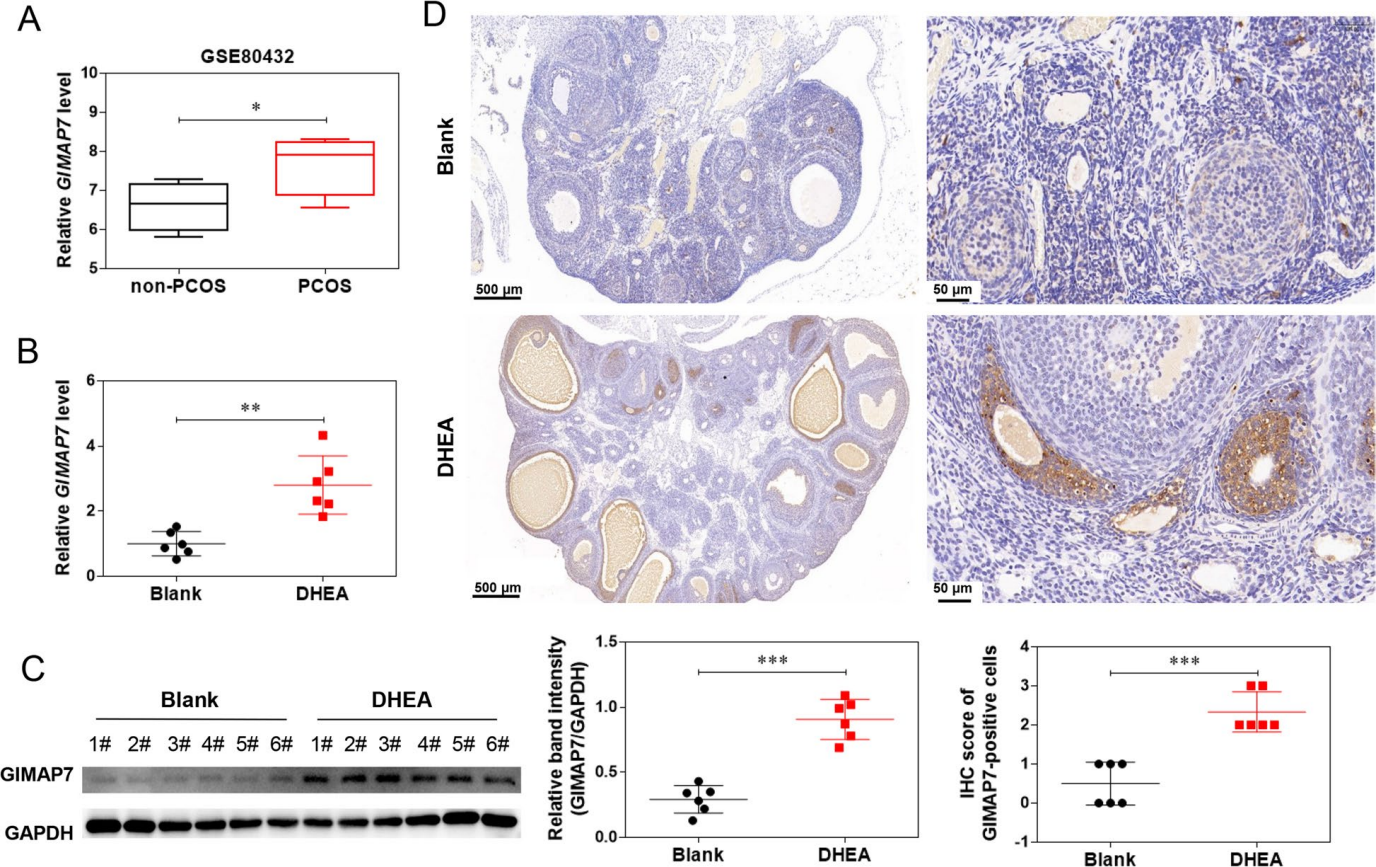
GIMAP7 expression is upregulated in PCOS rat. **A** The GIMAP7 expression in patients without PCOS (*n* = 4) and with PCOS (*n* = 4) from GEO dataset GSE80432. B-D: Sprague–Dawley rats (*n* = 6 per group) were injected with DHEA for constructing PCOS animal models. The rats in the blank group were injected with same volume of sesame oil. GIMAP7 mRNA (**B**) and protein (**C**) expression in ovarian tissues was measured using qRT-PCR and western blotting. **D** GIMAP7 expression in ovarian tissues was measured using IHC staining. Differences between two groups were analysed using Student’s t-test. *P < 0.05, **P < 0.01, ***P < 0.001

The GEO dataset GSE80432 revealed that GIMAP7 was upregulated in patients with PCOS compared to that in patients without PCOS (Fig. 1A). GIMAP7 expression in the ovarian tissues of DHEA-induced PCOS rats was assessed by qRT-PCR, western blotting, and IHC. The expression level of GIMAP7 was significantly higher in the DHEA group than in the blank group (Fig. 1B and C). IHC staining showed that GIMAP7 was mainly located in granulosa cells and was abundantly expressed in the DHEA group compared with the blank group (Fig. 1D).

### GIMAP7 shRNA relieves the symptoms of PCOS rats

**Fig. 2:**
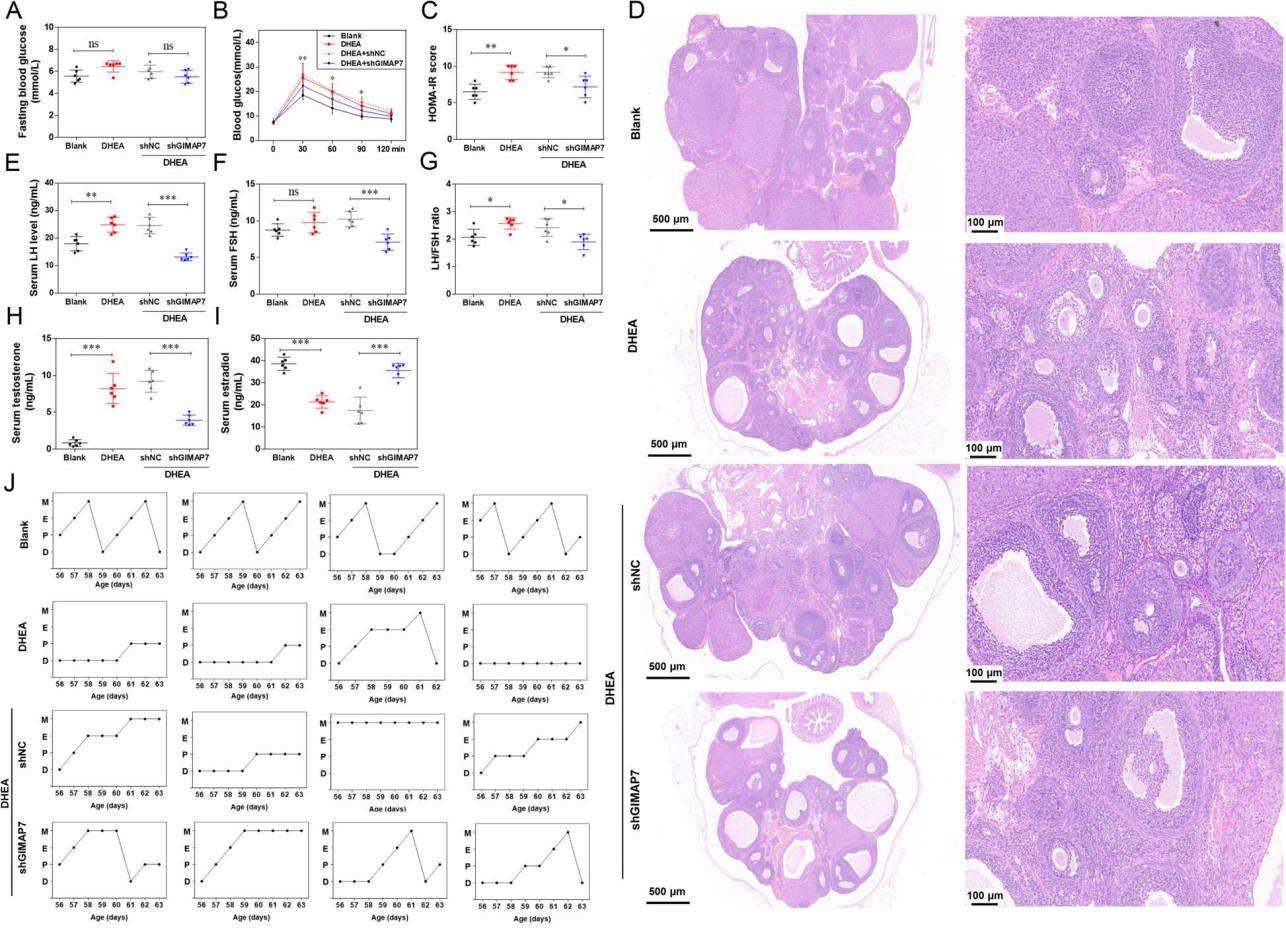
GIMAP7 shRNA relieves the symptoms of PCOS rats. **A**-**H** Sprague–Dawley rats (*n* = 6 per group) were injected with DHEA for constructing PCOS animal models, and then injected with lentivirus carrying shNC or shGIMAP7. The fasting blood-glucose (**A**), insulin tolerance tests (ITTs) (**B**), and homeostasis model assessment-insulin resistance (HOMA-IR) scores (**C**) were analysed in rats of each group. **D** Representative images of H&E staining show the histopathological changes of ovarian tissues. The serum LH (**E**), FSH (**F**), LH/FSH ratio (**G**), testosterone (**H**), and estradiol (**I**) were analysed using ELISA kits. **J** The representative oestrous cycles (D, dioestrus; P, proestrus; E, 0estrus; M, metestrus). Differences among multiple groups were analysed using a one-way analysis of variance. *P < 0.05, **P < 0.01, ***P < 0.001

To investigate the possible function of GIMAP7 in PCOS, GIMAP7 shRNA was injected into the ovaries of DHEA-induced PCOS rats. No significant differences were found in the fasting blood glucose levels among the four groups (Fig. 2A). The glucose levels and HOMA-IR scores in the DHEA group were significantly higher than those in the blank group (Fig. 2B and C). GIMAP7 shRNA significantly alleviated the abnormal glucose levels and HOMA-IR scores induced by DHEA. H&E staining showed an increase in atretic follicles (2.22 ± 0.45 vs. 6.5 ± 0.52) with fewer granulosa cell layers and increased follicular walls in DHEA rats as compared to the blank rats (Fig. 2D). As compared to shNC, GIMAP7 shRNA significantly decreased the number of atretic follicles (6.7 ± 0.43 vs. 3.4 ± 0.32). The serum levels of LH and FSH and the LH/FSH ratio were markedly increased in PCOS rats (Fig. 2E-G). GIMAP shRNA significantly decreased the levels of LH and FSH and reduced the LH/FSH ratio in the serum. Testosterone levels were increased while estradiol levels were decreased in the serum of PCOS rats; however, the increased testosterone levels and decreased estradiol levels in serum induced by PCOS were weakened by GIMAP shRNA (Fig. 2H-I). In addition, DHEA modelling disrupted the normal oestrous cycles, and most of the DHEA rats remained in the oestrus or dioestrus period (Fig. 2J). GIMAP7 shRNA largely corrected erratic oestrous cycles.

### GIMAP7 shRNA relieves ovarian apoptosis and oxidative stress in PCOS rats

**Fig. 3:**
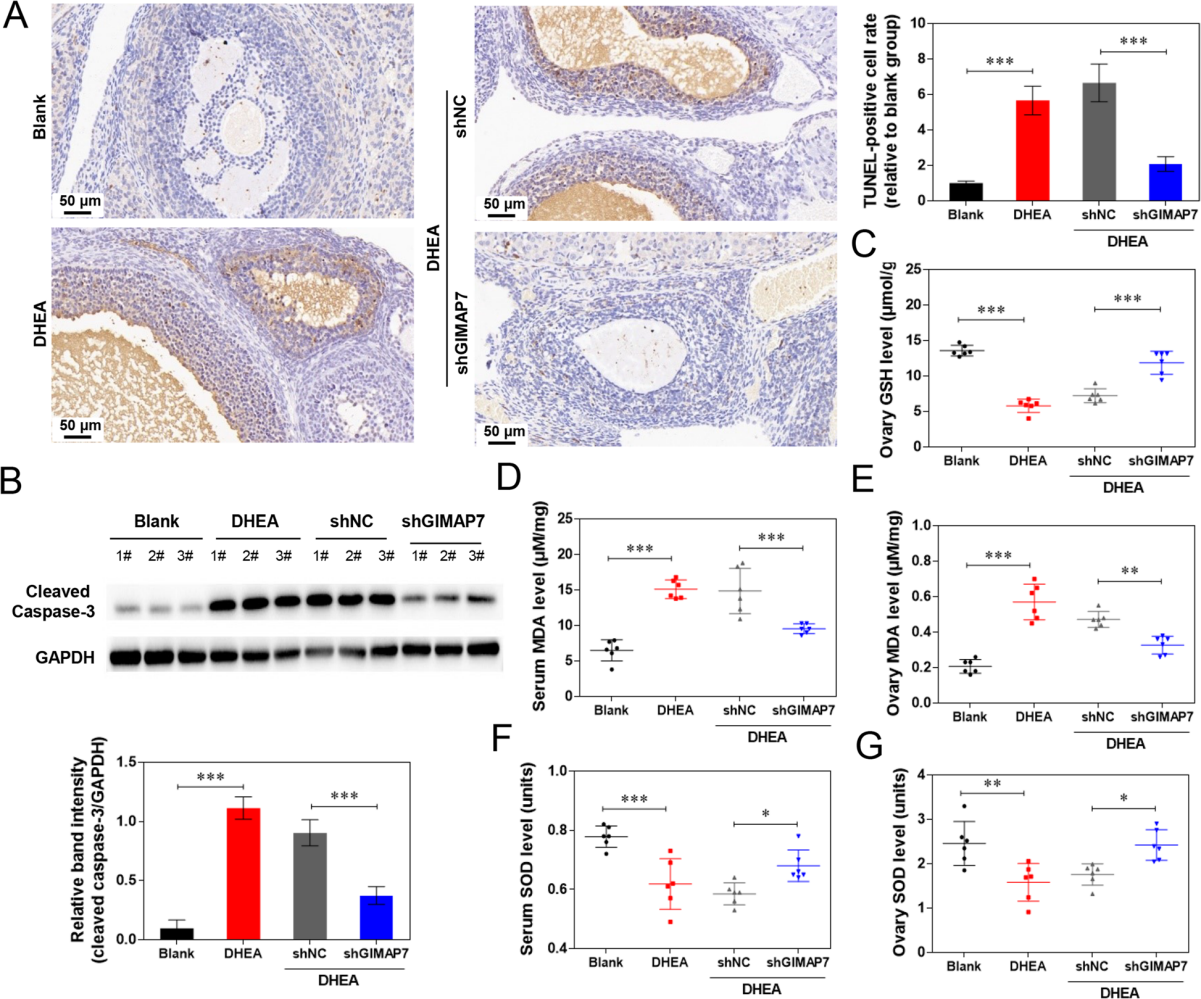
GIMAP7 shRNA relieves ovarian apoptosis and oxidative stress in PCOS rats. **A**-**F** Sprague–Dawley rats (*n* = 6 per group) were injected with DHEA for constructing PCOS animal models, and then injected with lentivirus carrying shNC or shGIMAP7. **A** The apoptosis of ovarian tissues was detected using TUNEL staining. Representative images of TUNEL are shown. **B** The protein expression of c-caspase-3 was detected using western blot. The GSH levels in ovaries (**C**). The MDA levels in serum (**D**) and ovaries (**E**). The SOD levels in serum (**F**) and ovaries (**G**). Differences among multiple groups were analysed using a one-way analysis of variance.*P < 0.05, **P < 0.01, ***P < 0.001

Apoptosis of the ovary and the apoptosis-related protein c-caspase-3 were detected by TUNEL staining and western blotting. DHEA modelling markedly increased the number of TUNEL-positive cells and enhanced the protein expression of c-caspase-3 compared with the blank group (Fig. 3A and B). GIMAP7 shRNA significantly decreased the number of TUNEL-positive cells and suppressed c-caspase-3 protein expression. Compared with the blank group, DHEA modelling significantly decreased GSH levels in the ovary; whilst GIMAP7 shRNA increased GSH levels (Fig. 3C). In addition, DHEA modelling significantly increased the MDA levels in the serum and ovary but decreased the SOD levels (Fig. 3D-G). GIMAP7 shRNA reduced the MDA levels and increased the SOD levels in the serum and ovaries compared to those in the shNC group.

### GIMAP7 siRNA promotes proliferation of ovarian granulosa cell line KGN

**Fig. 4:**
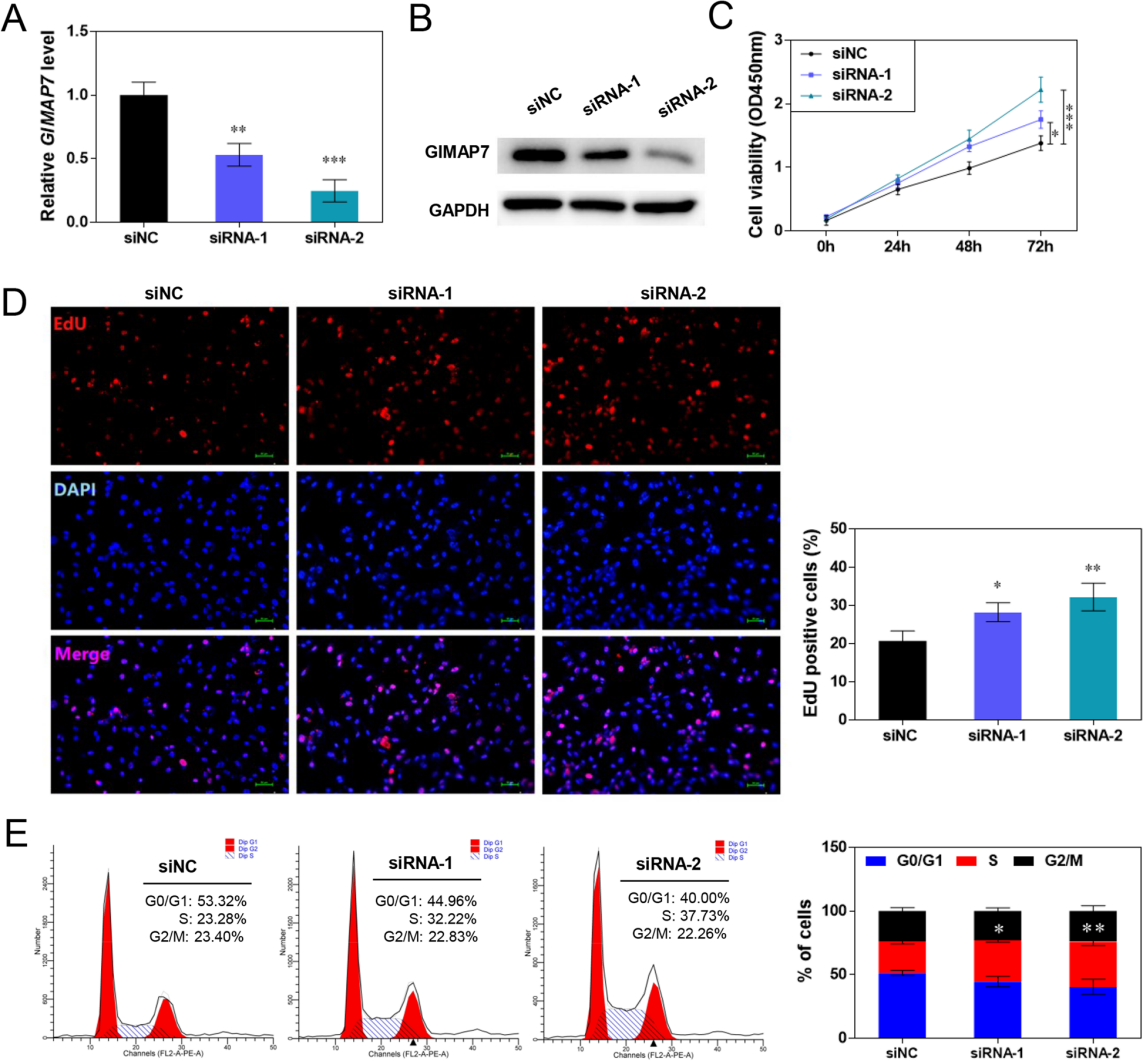
GIMAP7 siRNA promotes the proliferation of ovarian granulosa cells. **A**-**E** Ovarian granulosa cell line KGN was transfected with siNC or GIMAP7 siRNAs. The GIMAP7 mRNA (**A**) and protein (**B**) expression in KGN cells was detected using qRT-PCR and western blotting. The cell viability (**C**), proliferation (**D**), and cell cycle (**E**) were detected using CCK-8 assay, EdU staining, and flow cytometry. All experiments repeated three times. Images show only one representative result. Differences among multiple groups were analysed using a one-way or two-way analysis of variance. *P < 0.05, **P < 0.01, ***P < 0.001

An in vitro study was conducted in the ovarian granulosa cell line KGN to confirm the effects of GIMAP7 on PCOS. As shown in Fig. 4A and B, the expression of GIMAP7 in KGN cells was markedly decreased by GIMAP7 siRNAs. GIMAP7 siRNAs significantly increased the viability of KGN cells (Fig. 4C). The number of EdU-positive cells in the siRNA group was higher than that in the siNC group (Fig. 4D). In addition, GIMAP7 siRNAs markedly increased the proportion of KGN cells in S-phase (Fig. 4E).

### GIMAP7 siRNA inhibits the apoptosis and oxidative stress of KGN cells

**Fig. 5:**
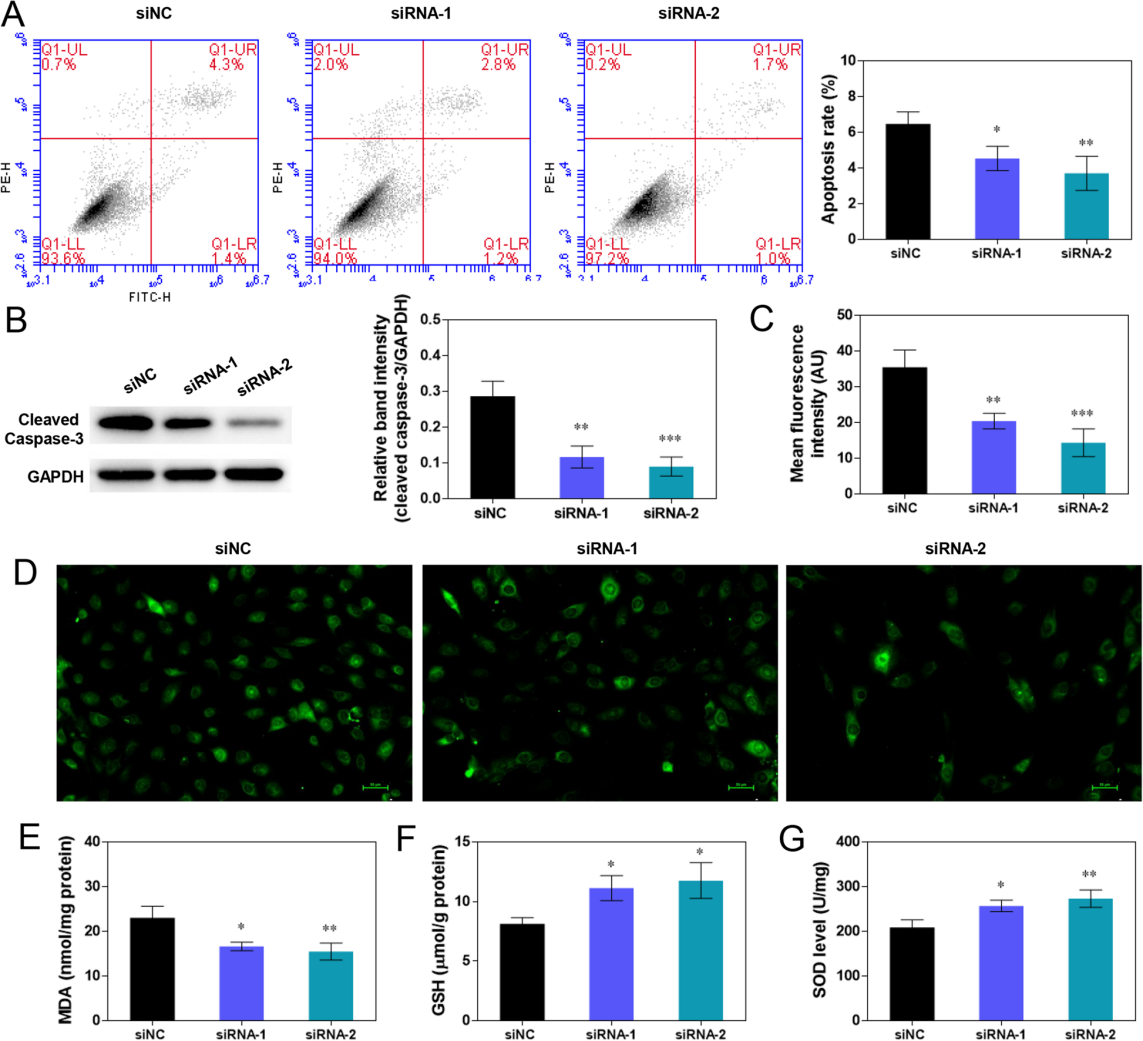
GIMAP7 siRNA inhibits the apoptosis and oxidative stress of KGN cells. **A**-**G** Ovarian granulosa cell line KGN was transfected with siNC or GIMAP7 siRNAs. **A** The KGN cell apoptosis was detected using flow cytometry. **B** The c-caspase-3 protein of KGN cells were measured using western blotting. The oxidative stress biomarkers ROS (**C**, **D**), MDA (**E**), GSH (**F**), and SOD (**G**) in KGN cells. All experiments repeated three times. Images show only one representative result. Differences among multiple groups were analysed using a one-way analysis of variance. *P < 0.05, **P < 0.01, ***P < 0.001

The data in Fig. 5A and B show that GIMAP7 siRNAs markedly inhibited apoptosis and decreased c-caspase-3 protein expression in KGN cells. Compared with the siNC group, the GIMAP7 siRNA groups showed weaker green fluorescence, indicating decreased ROS levels (Fig. 5C and D). The MDA levels were markedly decreased by GIMAP7 siRNAs (Fig. 5E). In addition, the levels of GSH and SOD were significantly increased by GIMAP7 siRNAs (Fig. 5F and G).

### GIMAP7 inhibits SHH signalling pathway in KGN cells

**Fig. 6:**
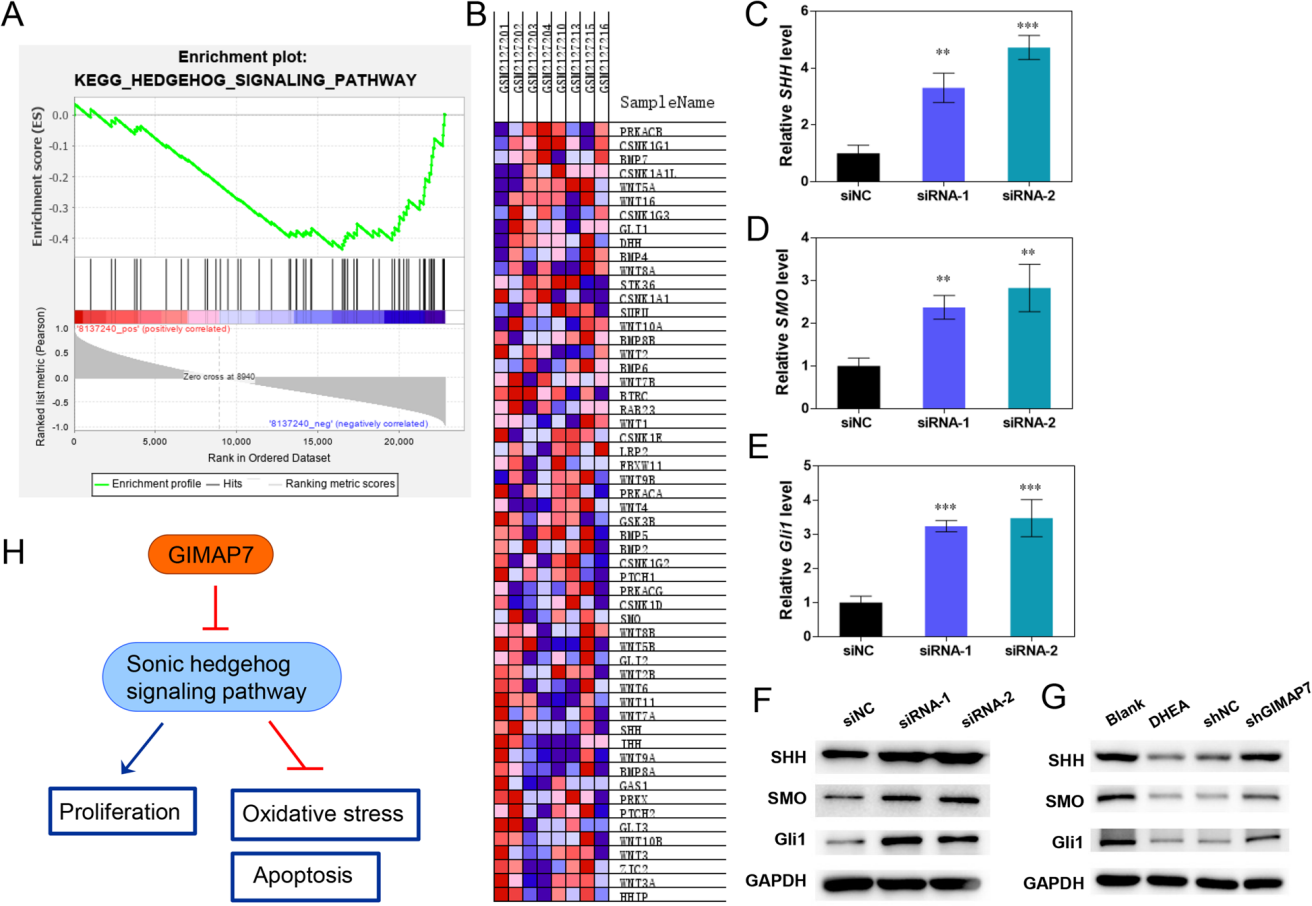
GIMAP7 inhibits the sonic hedgehog signalling pathway in KGN cells. **A** KEGG analysis showed that GIMAP7 inhibited the sonic hedgehog signalling pathway. **B** The genes enriched in the sonic hedgehog signalling pathway. **C**-**F** Ovarian granulosa cell line KGN was transfected with siNC or GIMAP7 siRNAs. The mRNA expression of *SHH* (**C**), *SMO* (**D**), and *Gli1* (**E**) in KGN cells was detected using qRT-PCR. **F** The protein expression of SHH, SMO, and Gli1 in KGN cells was detected using western blotting. **G** Sprague–Dawley rats were injected with DHEA for constructing PCOS animal models, and then injected with lentivirus carrying shNC or shGIMAP7. The protein expression of SHH, SMO, and Gli1 was detected using western blotting. **H** Diagram of GIMAP7 inhibiting the sonic hedgehog signalling pathway to promote PCOS progress. All experiments repeated three times. Images show only one representative result. Differences among multiple groups were analysed using a one-way analysis of variance. **P < 0.01, ***P < 0.001

To explore the potential mechanism of GIMAP7 in PCOS, KEGG was used to analyse the possible pathways regulated by GIMAP7. The results showed that GIMAP7 inhibited the SHH signalling pathway (Fig. 6A). Genes enriched in the SHH signalling pathway are shown in Fig. 6B. The expression of the SHH signalling pathway-related genes *SHH*, *SMO*, and *Gli1* was detected by qRT-PCR. GIMAP7 siRNAs dramatically increased the mRNA levels of *SHH*, *SMO*, and *Gli1* in KGN cells (Fig. 6C-E). Similar results were obtained for SHH, SMO, and Gli1 expression in KGN cells (Fig. 6F). In addition, SHH, SMO, and Gli1 were downregulated in the ovarian tissues of DHEA-induced rats, whereas they were upregulated in rats injected with lentivirus-carrying shGIMAP7 (Fig. 6G). A diagram of the effect of GIMAP7 on PCOS pathogenesis is shown in Fig. 6H. GIMAP7 inhibits the activation of the SHH signalling pathway in KGN cells, leading to inhibition of proliferation, cell oxidative stress, and apoptosis.

### Inhibition of SHH signalling pathway reduced the effects of GIMAP7 silencing on KGN cells

**Fig. 7:**
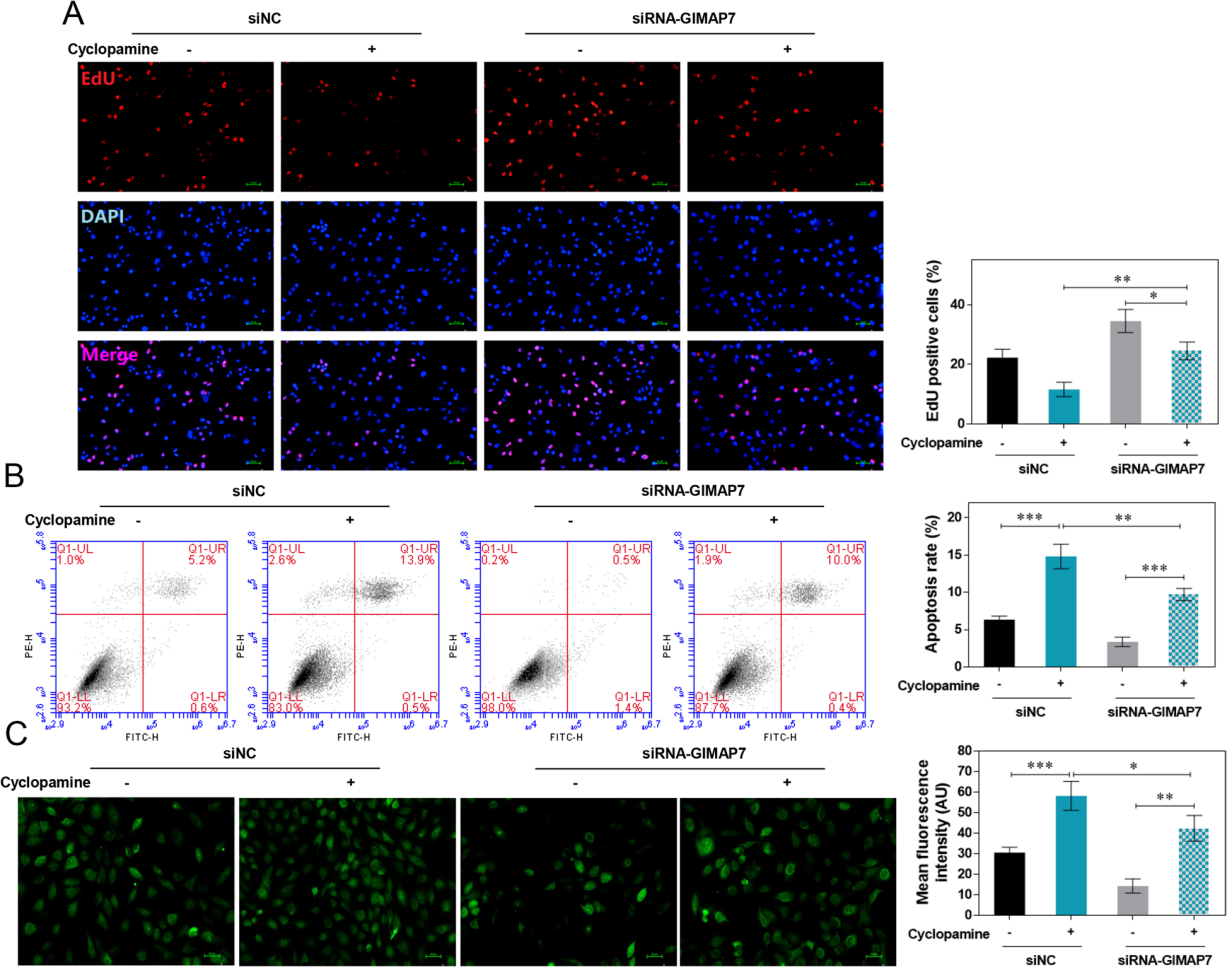
Inhibition of the SHH signalling pathway reduced the effects of GIMAP7 silencing on KGN cells. **A**-**C** Ovarian granulosa cell line KGN was transfected with siNC or GIMAP7 siRNAs, and were treated with 5 μM cyclopamine for 24 h. The proliferation (**A**), apoptosis (**B**), and ROS level (**C**) of KGN cells were detected using EdU staining, flow cytometry, and DCFDA staining. All experiments repeated three times. Images show only one representative result. Differences among multiple groups were analysed using a one-way analysis of variance.*P < 0.05, **P < 0.01, ***P < 0.001

Cyclopamine, an antagonist of the SHH signalling pathway, was employed to verify the regulatory effect of GIMAP7 on the SHH signalling pathway in KGN cells. The percentage of EdU-positive cells was markedly decreased by cyclopamine treatment, but increased by GIMAP7 siRNAs (Fig. 7A). Notably, the promoting effect of GIMAP7 siRNAs on KGN cell proliferation was neutralised by cyclopamine. The apoptosis rate of KGN cells was dramatically inhibited by GIMAP7 siRNA but increased by cyclopamine (Fig. 7B). The inhibitory effect of GIMAP7 siRNAs on the apoptosis of KGN cells was offset by cyclopamine. Additionally, GIMAP7 siRNAs decreased ROS levels, resulting in weaker green fluorescence (Fig. 7C). Cyclopamine significantly increased ROS levels, showing a brighter, weaker green fluorescence. It is worth noting that cyclopamine reduced the inhibitory effect of GIMAP7 siRNAs on ROS levels in KGN cells.

## Discussion

In this study, we demonstrated that GIMAP7 is upregulated in PCOS. GIMAP7 silencing promotes proliferation, inhibits apoptosis, and suppresses oxidative stress in ovarian tissues and granulosa cells. Furthermore, this study confirmed that GIMAP7 negatively regulated the SHH pathway. GIMAP7 exerted its effects on ovarian granulosa cell proliferation, apoptosis, and oxidative stress possibly via inhibition of SHH pathway.

PCOS is a common disease caused by complex endocrine and metabolic disorders in women of childbearing age, accompanied by insulin resistance and impaired glucose tolerance [23–25]. In this study, blood glucose levels and HOMA-IR scores were significantly increased in PCOS rats. The ovarian tissues showed increased atretic follicles with fewer granulosa cell layers and increased follicular walls in DHEA-injected rats. The serum levels of LH and FSH, and the LH/FSH ratio were significantly increased by DHEA. In addition, DHEA injection disrupted the normal oestrous cycles. GIMAP7 shRNA significantly alleviated the abnormality of energy metabolism and decreased the levels of LH and FSH, and corrected the erratic oestrous cycles of PCOS rats. These above results indicate that GIMAP7 may play a negative role in PCOS.

The imbalance caused by excessive oxidant formation (such as ROS) and limited antioxidant defence is defined as oxidative stress [26–28]; and markers of oxidative stress include SOD, GSH, and MDA [29]. Oxidative stress is considered a potential cause of PCOS [2, 29]. Studies have shown an excessive oxidative stress index in patients with PCOS and animal models [30–32]. In this study, DHEA injection significantly enhanced the oxidative stress, which was manifested as increased MDA levels and decreased SOD levels in the serum and ovaries. GIMAP7 silencing markedly decreased MDA levels and increased SOD levels in the serum and ovaries of PCOS rats. In KGN cells, GIMAP7 silencing significantly decreased ROS and MDA levels and increased GSH and SOD levels. In addition, GIMAP7 silencing increased cell viability, promoted proliferation, and increased the percentage of KGN cells in the S phase. These results indicated that GIMAP7 silencing alleviates PCOS by suppressing oxidative stress.

SHH is a member of the HH protein family and plays an important role in several diseases [33–35]. The SHH signalling pathway contains two ligands, smoothened (SMO) and 12-transmembrane receptors patched (Ptch), and the downstream target genes, cubitus interruptus transcription factor (Gli family). The release of SMO leads to the expression of Gli1 and Ptch by activating the Gli transcription factor [36]. Takebe et al. reported that the SHH signalling pathway promotes chondrogenesis during fracture healing in mice [37]. In cortical contusion injury mouse models, activation of the SHH signalling pathway significantly improves the recovery of motor function [38]. Downregulation of SHH aggravates disruption of the blood-brain barrier in mice with traumatic brain injury [39]. More importantly, SHH is observed in the ovary, granulosa cells, and oocytes, and promotes oocyte maturation in caprine [40]. Inhibition of the SHH signalling pathway intensified the injury to granulosa cells induced by hydrogen peroxide [22]. We found that the SHH signalling pathway was suppressed by GIMAP7. GIMAP7 siRNAs significantly increased the expression of SHH, SMO, and Gli1 in KGN cells. Additionally, cyclopamine, an antagonist of the SHH signalling pathway, markedly suppressed proliferation, promoted apoptosis and ROS generation, and weakened the protective effect of GIMAP7 on KGN cells. These data indicate that GIMAP7 silencing may alleviate PCOS by activating the SHH signalling pathway.

In conclusion, this study demonstrated that GIMAP7 is upregulated in PCOS and that GIMAP7 may promote apoptosis and oxidative stress by inhibiting the SHH signalling pathway. Thus, GIMAP7 may be a potential for PCOS therapy.

## Supplementary Information

**Additional file 1.**

## Data Availability

The datasets used and analyzed during the current study are available from the corresponding author on reasonable request.

## References

[1] Teede HJ, Misso ML, Costello MF, Dokras A, Laven J, Moran L, Piltonen T, Norman RJ (2018). Recommendations from the international evidence-based guideline for the assessment and management of polycystic ovary syndrome. Hum Reprod.

[2] Joksimovic Jovic J, Sretenovic J, Jovic N, Rudic J, Zivkovic V, Srejovic I, Mihajlovic K, Draginic N, Andjic M, Milinkovic M, Milosavljevic Z, Jakovljevic V (2021). Cardiovascular properties of the androgen-induced PCOS model in rats: the role of oxidative stress. Oxidative Med Cell Longev.

[3] Stein IF, Leventhal ML (1935). Amenorrhea associated with bilateral polycystic ovaries. Am J Obstet Gynecol.

[4] Sirmans S, Pate K (2013). Epidemiology, diagnosis, and management of polycystic ovary syndrome. Clin Epidemiol.

[5] Zheng Q, Li Y, Zhang D, Cui X, Dai K, Yang Y, Liu S, Tan J, Yan Q (2017). ANP promotes proliferation and inhibits apoptosis of ovarian granulosa cells by NPRA/PGRMC1/EGFR complex and improves ovary functions of PCOS rats. Cell Death Dis.

[6] Cappelli V, Musacchio M, Bulfoni A, Morgante G, Leo V (2017). Natural molecules for the therapy of hyperandrogenism and metabolic disorders in PCOS. Eur Rev Med Pharmacol Sci.

[7] Wikiera B, Zubkiewicz-Kucharska A, Nocoń-Bohusz J, Noczyńska A (2017). Metabolic disorders in polycystic ovary syndrome. Pediatric Endocrinol Diabetes Metab.

[8] Escobar-Morreale HF (2018). Polycystic ovary syndrome: definition, aetiology, diagnosis and treatment. Nat Rev Endocrinol.

[9] Kuşçu GC, Gürel Ç, Buhur A, Oltulu F, Akman L, Köse T, Yavaşoğlu NÜK, Yavaşoğlu A (2022). The regulatory effects of clomiphene and tamoxifen on mTOR and LC3-II expressions in relation to autophagy in experimental polycystic ovary syndrome (PCOS). Mol Biol Rep.

[10] Ruan X, Kubba A, Aguilar A, Mueck AO (2017). Use of cyproterone acetate/ethinylestradiol in polycystic ovary syndrome: rationale and practical aspects. Eur J Contracept Reprod Health Care.

[11] Krücken J, Schroetel RMU, Müller IU, Saïdani N, Marinovski P, Benten WPM, Stamm O, Wunderlich F (2004). Comparative analysis of the human gimap gene cluster encoding a novel GTPase family. Gene.

[12] Rutledge EA, Fuller JM, Van Yserloo B, Moralejo DH, Ettinger RA, Gaur P, Hoehna JL, Peterson MR, Jensen R, Kwitek AE, Lernmark Å (2009). Sequence variation and expression of the <i>Gimap</i> gene family in the BB rat. Exp Diabetes Res.

[13] Schnell S, Démollière C, van den Berk P, Jacobs H (2006). Gimap4 accelerates T-cell death. Blood.

[14] Qin Y, Liu H, Huang X, Huang L, Liao L, Li J, Zhang L, Li W, Yang J (2022). GIMAP7 as a potential predictive marker for Pan-Cancer prognosis and immunotherapy efficacy. J Inflamm Res.

[15] Huo X, Shen G, Li J, Wang M, Xie Q, Zhao F, Ren D, Dong Q, Zhao J (2022). Identification of the GTPase IMAP family as an immune-related prognostic biomarker in the breast cancer tumor microenvironment. Gene.

[16] Usman M, Ilyas A, Hashim Z, Zarina S (2020). Identification of GIMAP7 and Rabl3 as putative biomarkers for Oral squamous cell carcinoma through comparative proteomic approach. Pathol Oncol Res.

[17] Guo C, Tang Y, Zhang Y, Li G. Mining TCGA data for Key biomarkers related to immune microenvironment in endometrial cancer by immune score and weighted correlation network analysis. Front Mol Biosci. 2021;8:645388 10.3389/fmolb.2021.645388 PMC8048410 33869285

[18] Song Y, Pan Y, Liu J (2019). The relevance between the immune response-related gene module and clinical traits in head and neck squamous cell carcinoma. Cancer Manag Res.

[19] Meng Z, Ren D, Zhang K, Zhao J, Jin X, Wu H (2020). Using ESTIMATE algorithm to establish an 8-mRNA signature prognosis prediction system and identify immunocyte infiltration-related genes in pancreatic adenocarcinoma. Aging.

[20] Moralejo DH, Fuller JM, Rutledge EA, Van Yserloo B, Ettinger RA, Jensen R, Osborne W, Kwitek A, Lernmark Å (2011). BB rat Gimap gene expression in sorted lymphoid T and B cells. Life Sci.

[21] Au-McLean AC, Au-Valenzuela N, Au-Fai S, Au-Bennett SAL (2012). Performing vaginal lavage, crystal violet staining, and vaginal cytological evaluation for mouse estrous cycle staging identification. JoVE.

[22] Wang X, Fan G, Wei F, Bu Y, Huang W (2019). Hyperoside protects rat ovarian granulosa cells against hydrogen peroxide-induced injury by sonic hedgehog signaling pathway. Chem Biol Interact.

[23] Diamanti-Kandarakis E, Kouli C, Bergiele A, Filandra F, Tsianateli T, Spina G, Zapanti E, Bartzis M (1999). A survey of the polycystic ovary syndrome in the Greek Island of Lesbos: hormonal and metabolic profile. J Clin Endocrinol Metab.

[24] Azziz R, Woods K, Reyna R, Key T, Knochenhauer E, Yildiz B (2004). The prevalence and features of the polycystic ovary syndrome in an unselected population. J Clin Endocrinol Metab.

[25] Legro R, Arslanian S, Ehrmann D, Hoeger K, Murad MH, Pasquali R, et al. Diagnosis and treatment of polycystic ovary syndrome: an Endocrine Society clinical practice guideline. J Clin Endocrinol Metab. 2013;98:4565–92. 10.1210/jc.2013-2350 PMC5399492 24151290

[26] Estévez M (2015). Oxidative damage to poultry: from farm to fork. Poult Sci.

[27] Turrens JF (2003). Mitochondrial formation of reactive oxygen species. J Physiol.

[28] Mohammadi M (2019). Oxidative stress and polycystic ovary syndrome: a brief review. Int J Prev Med.

[29] Murri M, Luque-Ramírez M, Insenser M, Ojeda-Ojeda M, Escobar-Morreale HF (2013). Circulating markers of oxidative stress and polycystic ovary syndrome (PCOS): a systematic review and meta-analysis. Hum Reprod Update.

[30] Liu Y, Yu Z, Zhao S, Cheng L, Man Y, Gao X, Zhao H (2021). Oxidative stress markers in the follicular fluid of patients with polycystic ovary syndrome correlate with a decrease in embryo quality. J Assist Reprod Genet.

[31] Sun Y, Li S, Liu H, Bai H, Hu K, Zhang R, Liu Q, Fan P (2021). Oxidative stress promotes hyperandrogenism by reducing sex hormone-binding globulin in polycystic ovary syndrome. Fertil Steril.

[32] Ebrahimi F, Rostami S, Nekoonam S, Rashidi Z, Sobhani A, Amidi F (2021). The effect of Astaxanthin and metformin on oxidative stress in Granulosa cells of BALB C mouse model of polycystic ovary syndrome. Reprod Sci.

[33] Goetz JA, Suber LM, Zeng X, Robbins DJ (2002). Sonic hedgehog as a mediator of long-range signaling. Bioessays.

[34] Hosoya A, Shalehin N, Takebe H, Shimo T, Irie K. Sonic hedgehog signaling and tooth development. Int J Mol Sci. 2020;21(5):1587. 10.3390/ijms21051587 PMC7084732 32111038

[35] Qin Y, Jiang M, Tuerxung N, Wang H, Zhao F, Zhen Y, Hao J (2020). Sonic hedgehog signaling pathway in Myelodysplastic syndrome: abnormal activation and jervine intervention. Gene.

[36] Wang XZ, Zhang HH, Qian YL, Tang LF (2019). Sonic hedgehog (Shh) and CC chemokine ligand 2 signaling pathways in asthma. J Chin Med Assoc.

[37] Takebe H, Shalehin N, Hosoya A, Shimo T, Irie K. Sonic hedgehog regulates bone fracture healing. Int J Mol Sci. 2020;21(2):677. 10.3390/ijms21020677 PMC7013927 31968603

[38] Pringle AK, Solomon E, Coles BJ, Desousa BR, Shtaya A, Gajavelli S, Dabab N, Zaben MJ, Bulters DO, Bullock MR, Ahmed AI (2021). Sonic hedgehog signaling promotes Peri-lesion cell proliferation and functional improvement after cortical contusion injury. Neurotrauma Rep.

[39] Michinaga S, Inoue A, Sonoda K, Mizuguchi H, Koyama Y (2021). Down-regulation of astrocytic sonic hedgehog by activation of endothelin ET(B) receptors: involvement in traumatic brain injury-induced disruption of blood brain barrier in a mouse model. Neurochem Int.

[40] Wang DC, Huang JC, Lo NW, Chen LR, Mermillod P, Ma WL, Chiang HI, Ju JC (2017). Sonic hedgehog promotes in vitro oocyte maturation and term development of embryos in Taiwan native goats. Theriogenology.

