# Supplementary material for: GIMAP7 induces oxidative stress and apoptosis of ovarian granulosa cells in polycystic ovary syndrome by inhibiting sonic hedgehog signalling pathway
Source: J Ovarian Res. 2022 Dec 30;15:141. doi: 10.1186/s13048-022-01092-z (PMC9801623; doi:10.1186/s13048-022-01092-z)
Supplement: Supplementary file 1 — Additional file 1. [file 13048_2022_1092_MOESM1_ESM.docx]

**Figure 1D**

**GIMAP7**

**



**

**GAPDH**

**



**

**Figure 3B**

**Cleaved caspase-3**

**



**

**GAPDH**

**



**

**Figure 4B**

**GIMAP7**

**



**

**GAPDH**

**



**

**Figure 5B**

**Cleaved caspase-3**

**



**

**GAPDH**

**



**

**Figure 6F**

**SHH**

**



**

**SMO**

**



**

**Gli1**

**



**

**GAPDH**

**



**
